# Individual differences in the perception of biological motion and fragmented figures are not correlated

**DOI:** 10.3389/fpsyg.2013.00795

**Published:** 2013-10-30

**Authors:** Eunice L. Jung, Asieh Zadbood, Sang-Hun Lee, Andrew J. Tomarken, Randolph Blake

**Affiliations:** ^1^Department of Brain and Cognitive Sciences, Seoul National UniversitySeoul, South Korea; ^2^Department of Psychology, Vanderbilt UniversityNashville, TN, USA

**Keywords:** perceptual grouping, biological motion, fragmented figures, individual differences

## Abstract

We live in a cluttered, dynamic visual environment that poses a challenge for the visual system: for objects, including those that move about, to be perceived, information specifying those objects must be integrated over space and over time. Does a single, omnibus mechanism perform this grouping operation, or does grouping depend on separate processes specialized for different feature aspects of the object? To address this question, we tested a large group of healthy young adults on their abilities to perceive static fragmented figures embedded in noise and to perceive dynamic point-light biological motion figures embedded in dynamic noise. There were indeed substantial individual differences in performance on both tasks, but none of the statistical tests we applied to this data set uncovered a significant correlation between those performance measures. These results suggest that the two tasks, despite their superficial similarity, require different segmentation and grouping processes that are largely unrelated to one another. Whether those processes are embodied in distinct neural mechanisms remains an open question.

## Introduction

**Figure 1:**
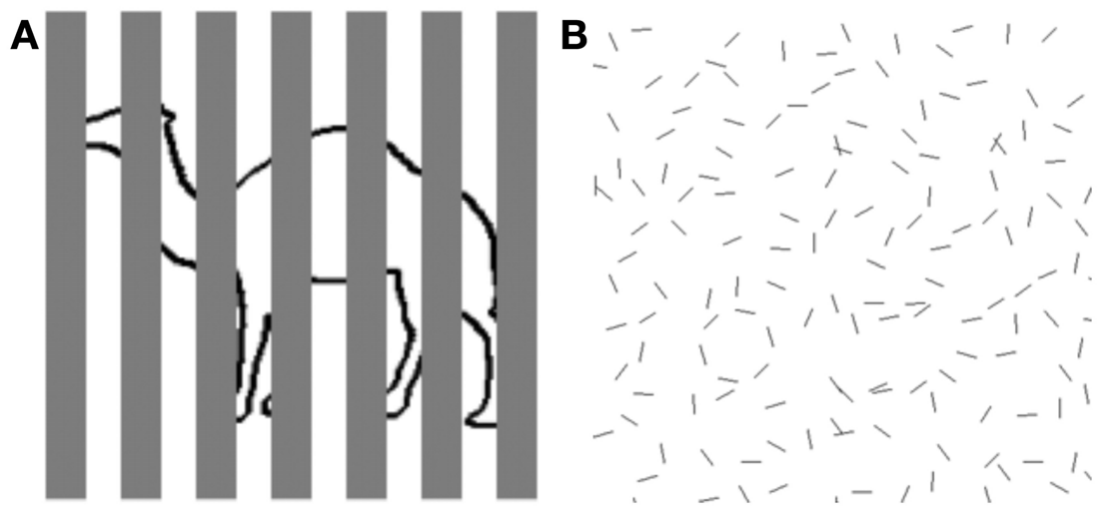
**Examples of object perception in cluttered scenes. (A)** Fragmented figure. Although approximately half of the contour defining the outline object is occluded from view, we can readily tell that it is a camel. Speed and accuracy of object recognition are directly related to the proportion of visible object contour. **(B)** Figure ground segregation. The hexagon-shaped figure defined by spatial structure (good continuation) can be located within a cluttered background of noise contours identical in shape to the individual contours comprising the hexagon. Speed and accuracy of figure detection are inversely related to the density of noise elements.

In our everyday lives, objects of interest to us are sometimes not so easy to see because of the cluttered visual context in which they appear. One source of this difficulty is created when our view of an object is partially obstructed by other, nearer objects, creating the condition known as partial occlusion (see Figure 1A). Another source of difficulty can arise when the object is highly similar to other neighboring objects in terms of shape and surface appearance, thereby complicating the process known as figure/ground segregation (see Figure 1B). These challenges to object perception have long been recognized by vision scientists (Von Helmholtz, 1866/1925), and the Gestalt psychologists famously proposed rules of perceptual organization that counteracted the fragmented, partially obscured nature of the optical input to vision (Wertheimer, 1938). Elaboration and refinement of those rules have continued to be the focus of much contemporary work on vision (e.g., Singh and Hoffman, 1998; Geisler et al., 2001; Frisby and Stone, 2010).

Our understanding of perceptual organization has benefitted greatly from the development of psychophysical strategies for isolating the contributions of specific operations putatively involved in object perception. One such strategy is exemplified by the fragmented figures technique popularized by Snodgrass et al. (1987). By creating these kinds of fragmented line drawings of objects, investigators can target the process of spatial integration whereby isolated local features are combined into a globally coherent figure. Fragmented figures allow one to assess quantitatively the effect of figure degradation on picture identification and to exploit figure degradation to study processes such as perceptual learning (Doniger et al., 2001a), visual priming (Snodgrass and Feenan, 1990), and implicit and explicit memory (Russo et al., 1995). Another popular stimulus strategy for studying perceptual organization involves the use of point-light (PL) animations to portray biological motion. With this technique, the hierarchical, pendular motions characterizing human body movements are portrayed using just a small number of points of light attached to the limbs, torso, and head of a human actor (Johansson, 1973) or, for that matter, attached to the limbs of a non-human vertebrate (Blake, 1993; Mather and West, 1993). When animated, the structured motions of the dots create the immediate, compelling impression of a body engaged in a specific activity (e.g., running), with sufficient fidelity to convey not only the type of activity but also the sex and emotional state of the actor (see review by Blake and Shiffrar, 2007). Biological motion portrayed by PL animation has been widely used in recent years to study dynamic perceptual organization in children (Pavlova et al., 2001; Friere et al., 2006), young adults (Hiris, 2007) and the elderly (Norman et al., 2004; Billino et al., 2008; Pilz et al., 2010), as well as in clinical populations including people with autism (Moore et al., 1997; Blake et al., 2003; Kaiser and Shiffrar, 2009; McKay et al., 2012; Nackaerts et al., 2012), prosopagnosia (Lange et al., 2009), schizophrenia (Kim et al., 2005, 2011; Spencer et al., 2013), and brain damage (Cowey and Vaina, 2000; Pavlova et al., 2003).

In the study reported here, we sought to learn whether grouping involved in perception of static, fragmented figures (FFs) is related to grouping involved in perception of dynamic figures portrayed by PL animations. For several reasons, this conjecture seemed plausible to us. First, while differing in the ways they are created, both fragmented figures and PL animations require integration of spatially distributed local elements in order to perceive the global shape of entire figures. It is true, of course, that discerning a biological figure from a single-frame snapshot of a PL animation can be very difficult (Johansson, 1973), suggesting that grouping in PL animations entails integration of local motion signals (e.g., Mather et al., 1992). Still, there is evidence suggesting that form information contributes to efficient processing of biological motion information contained in PL animations. For example, perception of biological motion is possible even when viewing PL animations in which frame-to-frame dot locations are repositioned on body parts other than the limbs (Beintema and Lappe, 2002), or when viewing animations where dots are presented with limited life-times (Beintema et al., 2006) or in configurations that disrupt normal structural regularities present during human locomotion (Pinto and Shiffrar, 1999). These maneuvers, it has been argued, should seriously impair perception of biological motion based exclusively on local motion, yet in practice the impairments are mild. For these and other reasons, most researchers now believe that motion and form interact at some point in processing to mediate perception of biological motion, a view that is embodied in recent computational models (e.g., for a review see Fleischer and Giese, 2012). Also consistent with this view are the widespread neural responses within both form- and motion-selective brain areas in people viewing PL animations (Grossman and Blake, 2002; Peelen et al., 2006; Jastorff and Orban, 2009; McKay et al., 2012; Thompson and Baccus, 2012). Finally, perceptual results from clinical neuropsychological studies hint at a possible linkage between the two forms of grouping. Specifically, one study shows that schizophrenic patients have trouble recognizing objects portrayed as fragmented figures (Doniger et al., 2001b) and several other studies have found that patients diagnosed with schizophrenia exhibit deficits in biological motion perception (Kim et al., 2005, 2011; Spencer et al., 2013).

So considered together, these various lines of evidence sparked our interest in studying perceptual grouping using FFs and PL animations. To pursue this question of the possible relation of task performance on these two forms of perceptual grouping, we utilized an individual differences approach (Kanai and Rees, 2011). We tested a large group of healthy, college-aged individuals on a set of psychophysical tasks designed to measure perceptual grouping performance using biological motion animations and fragmented figures. Our aim was to determine whether performance measures on the two tasks were correlated across observers. Anticipating the possibility that this correlation might indeed be statistically significant, we wanted to be able to evaluate whether that correlation might be attributable to individual differences in non-perceptual factors such as vigilance and/or motivation. So we also administered a third test to each participant, a contrast detection task that utilized a very simple stimulus, i.e., a gabor patch of fixed orientation and spatial frequency. Performance on this task, which putatively relies on neural events transpiring at very early stages of visual processing of elementary features, does not require perceptual grouping but does require sustained concentration, just like the tasks using fragmented figures and biological motion animations. For all three tasks we employed the same general design: a two-interval, forced-choice (2IFC) procedure in which task difficulty was varied over successive trials according to QUEST (Watson and Pelli, 1983), an adaptive staircase procedure that efficiently provides reliable estimates of visual thresholds. We employed 2IFC, rather than a Yes/No procedure, to minimize criterion differences (i.e., willingness to say “yes”) within our large sample of naive participants. We decided on the temporal forced-choice procedure rather than a spatial forced choice procedure because the PL animations used in our study included activities that are not cyclic (e.g., throwing) and, therefore have distinct start and end points. Thus, we had to avoid exposure durations longer than the action cycle (1-s) so as not to introduce abrupt transients associated with repetition of an animation, which may provide an unwanted cue for discrimination of a PL animation from a non-PL animation. With this brief exposure duration, it was impractical to use a spatial forced choice task wherein the observer would be required to shift her/his gaze successively to view the two alternatives. Nor we did not want to force the observer to maintain fixation midway between the two displays for that would have imaged the two displays in the visual periphery where acuity is poorer. For these reasons, we chose 2IFC. We realize that 2IFC procedures can be susceptible to response bias toward an interval with a particular order (Yeshurun et al., 2008), but that type of bias can be directly assessed from the 2IFC data.

## Methods

### Participants

A total of seventy-seven individuals participated in this study, which was performed at two institutions. At Seoul National University (SNU) in South Korea, fifty-seven healthy paid volunteers, aged 20–33 (mean age = 24.4 years) with normal or corrected-to normal vision, were recruited through an online posting. Approximately half of the participants were female (*n* = 27). One female subject was excluded from the data analysis because she misunderstood the task instructions, leaving us with a total of 76 data sets for analysis. The experimental procedures were approved by the Institutional Review Board of the Seoul National University, and each volunteer gave written consent to participate in the experiment. At the second site, Vanderbilt University (VU) in Nashville, TN, twenty healthy students participated in the experiment for course credit. This group consisted of seven male and thirteen female students aged 18–21 (mean age = 19.25 years). Each gave written consent to procedures as approved by the Institutional Review Board of Vanderbilt University.

At SNU, each participant was screened using the Optec 5000 vision tester (Stereo Optical Co., Inc), with refractive correction worn if needed. All participants exhibited stereoacuity values and near and far visual acuities within the normal range. Because the stimuli used in our study were grayscale, not color, we did not eliminate individuals from our study based on color vision even though four participants made a few mistakes on the color plates included in the test battery. None of the participants reported any history of reading problems or symptoms of abnormal vision. The VU participants were recruited through a web-based research sign-up system, and one of the criteria listed for participation was normal or corrected-to-normal acuity in both eyes.

### General task and procedure

Visual stimuli were generated using MATLAB (Mathworks Inc. Natick, MA) in conjunction with the Psychophysics Toolbox V3.0.9 (Brainard, 1997; Pelli, 1997; Kleiner et al., 2007) running on a Macintosh computer, and the stimuli were presented on a large screen video monitor (model LG 2363D for SNU, and a Totoku Calix model # CDT2141A for VU). Participants viewed the screen from a distance of 60 cm, with the head position stabilized comfortably on a chinrest. Monitor settings, viewing distance and ambient test-room illumination were identical for the two testing sites, as were the stimuli (described in the following section).

Participants performed three tasks: biological motion discrimination (BM), fragmented figures discrimination (FF), and gabor patch detection (GD). All three tasks were administered using a two-interval forced choice (2IFC) design. For the two discrimination tasks, one interval contained a target stimulus embedded in noise and the other interval contained a disorganized version of that target stimulus in noise. For both tasks the level of noise was varied over trials according to an adaptive procedure (QUEST) to derive 75% correct threshold estimates, and this procedure was repeated three times for each task. For the GD task, one interval contained a 1D gabor patch superimposed on a weak, 2D noise background and the other interval contained the noise background only. For this detection task, gabor patch contrast was varied over trials adaptively to find the 75% threshold, with the procedure repeated three times. For each of the three tasks, the QUEST parameters were optimized based on pilot experiments performed on a different group of participants from those tested in the main study. The entire visual stimuli, including the noise masks, for all three tasks encompassed a 10 × 10° display area.

Thus the formal test session consisted of 9 blocks of trials (3 tasks × 3 repetitions for each task), with each block consisting of 50 trials. Each trial consisted of two 1-s intervals separated by a 0.5 s blank screen between intervals. The order of target and non-target intervals was randomized over trials, and following each trial the participant indicated which interval contained the target by pressing one of two buttons on the keypad, guessing if necessary. Participants were under no speed constraint for responding, and error feedback was not given. A block of trials typically took about 4 to 5 min to complete, and participants were free to rest between trial blocks.

### Task 1: biological motion discrimination

#### Stimuli

The target stimuli for this task were point-light (PL) animations (Johansson, 1973) in which a small number of dots strategically placed on the limbs, torso and head of a human actor depict the kinematics of the activity (e.g., walking) of the actor. The details used for producing these particular PL sequences have been described elsewhere (Grossman and Blake, 2002). Our PL animations consisted of black dots appearing against a white background. The number of dots in each frame of an animation could range from 9 to 12 depending on whether the activity produced an occluded body part. In one of the animations (kicking) the torso dot was missing in all frames. When viewed at an animation frame rate appropriate for portraying the actual speed of human body movements, these PL sequences produce the vivid impressions of the actor's movements. Perception of biological motion from PL animations relies on extraction of the kinematics of the event conveyed by spatiotemporal integration of the changing positions of the dots, which is why adding dynamic noise dots to a PL animation can perturb perception of biological motion (e.g., Hiris et al., 2005).

For the present study we wanted to use a set of PL sequences that were matched as closely as possible in detectability when presented within noise. We knew from pilot work that some actions within our library of sequences were easier to discern than others when masking noise is used, and those differences in salience among sequences would inevitably produce unwanted trial to trial variability in task performance that, in turn, would adversely impact QUEST's ability to converge on a reliable estimate of threshold. So prior to our main experiment, we performed a pilot study in which we tested five undergraduate volunteers using a two-up/one-down staircase procedure to estimate performance threshold estimates using 15 different PL sequences in our library of biological motion sequences. We purposefully avoided including PL animations that are ambiguous with respect to the facing direction of the actor (Vanrie et al., 2004; Jackson and Blake, 2010), to minimize perceptual confusion and to avoid possible complications associated with individual differences in factors related to personality (Heenan and Troje, 2012).

**Figure 2:**
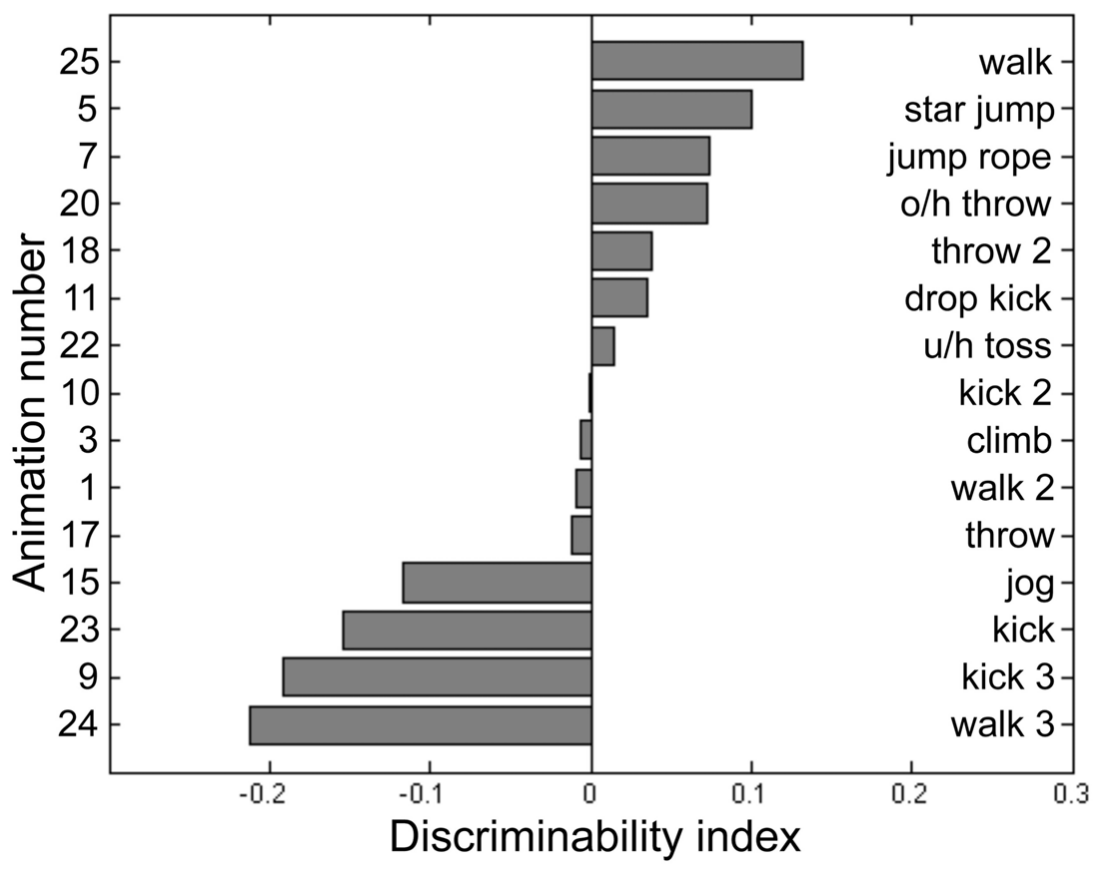
**Discriminability index calculated for 15 point-light (PL) animations each portraying a unique human activity (see text for details of data collection and analysis).** From this set of 15 we selected those four PL activities located closest to the median index value and used those four PL animations in the main experiment. The left side of the panel lists the animation number, and the right lists its associated activity name. O/h throw: overhead throw. U/h toss: underhand toss.

Participants for this pilot experiment performed 12 blocks of the BM task across 4 sessions. Each block consisted of 2IFC trials guided by a 2-up/1-down staircase procedure, which started at 20 noise dots for BM. The staircase was terminated after 16 reversals and the performance threshold was estimated by averaging the noise level present at the last 8 reversals. For BM, the noise dots increased by 6 dots per two successive correct trials for the first 12 reversals, then decreased to 3 dots per two successive correct trials in the remainder of the block sequence. Participants performed an average of 844 trials for this BM pilot task, the results from which were used to fit a cumulative normal distribution using the percent-correct values plotted against noise level. The purpose of this psychometric curve fitting was to derive parameters for a “model performance” for each participant at a given noise level, and to achieve this we pooled performance values across all 15 PL sequences and utilized only the noise level information (i.e., psychometric curve fitting was blind to the different PL sequences). Next, we calculated the average percent-correct associated with each PL sequence tested over a range of noise levels. From these data we derived an index of stimulus difficulty for each PL sequence, defined in terms of the difference between predicted performance fitted by the psychometric curve and the actual measured performance for each of the 15 PL sequences. We interpret this index of stimulus difficulty—i.e., the deviation from the model performance—as a reflection of those inherent characteristics of a given PL sequence governing its difficulty relative to all other sequences presented at a given noise level. The values of this index for each sequence were based on the pooled data for the five participants, and from the distribution of index values (see Figure 2) we selected the four PL sequences centered on the median of that distribution (meaning activities that were equally discriminable that were neither exceedingly difficult nor especially easy and that produced the smallest variability across repeated estimates). This procedure yielded the following four PL activities: climbing, dropkicking, overhead throwing, and underhand tossing (corresponding to animation number 3, 11, 20, 22, respectively, in Figure 2). Creating right-left flipped counterparts for each of these four motions thus provided us a total of 8 target stimuli for this task. Some dots (joints) could be occluded during a frame or two of the animation, but the brief disappearance of a dot was only conspicuous when the animation was viewed without noise. The actual angular subtense of a given PL sequence varied depending on the particular action being portrayed and depending on the point in time during the 1-s action sequence. The average angular subtense computed over all four action types and time frames was 1.8° (width) and 4.7° (height).

As mentioned above, a given PL animation appeared within one of two successive, 1-s presentation intervals on each trial of this 2IFC task. Within this 1-s interval, the movement went through approximately a complete action cycle, and the animation always started from the beginning of a cycle (which is necessary to avoid abrupt transitions that would be produced by varying the start-frame a non-cyclic action such as kicking). The actual location of a PL figure within the display was jittered from trial to trial relative to the fixation point at the center of the display, to discourage observers from monitoring a specific spatial location for some pattern of diagnostic dot motion. Each PL sequence could be jittered up to 2°, with the specific jitter value used on each trial being drawn from a normal distribution.

In the non-target interval, a 1-s animation depicting disorganized biological motion (DM) was created online (i.e., prior to each trial) from the target BM sequence used on a given trial. Our lab recently developed these DM animation sequences using a new procedure that differs from the traditional “position scrambling” procedure used by some in previous work (e.g., Grossman et al., 2010). As in the traditional procedure, we relocated the dots' starting positions over space. However, we put several constraints on the way the starting positions were redistributed. These constraints were introduced to minimize two unwanted cues that potentially contribute to discrimination of an original, intact BM sequence from its “position scrambled” version, wherein the starting positions of the individual dots are randomized independently of each other. First, this independent, random repositioning technique, although effectively destroying the global coherent structure of an original sequence, invites substantial changes in motion energy composition. Random and independent repositioning of dots guarantees that the motion trajectories of the individual twelve dots are retained, but it has no control over motion energy arising from interactions among those twelve dots. This allows observers to discriminate any given intact BM sequence against its scrambled version also based on motion energy differences, not only based on global motion coherence unique to biological motion, which is the cue that we intend to selectively impair for the non-target interval stimulus in the current study. Second, the unconstrained randomization of individual dots' starting positions results in substantial changes in spatial distribution of dots in any given single frame of an animation, thus providing an additional cue for discrimination. For example, observers might rely on an impression of “spatial sparseness/density” to judge whether an animation sequence in a given interval is a target or non-target. It is desirable to remove or minimize the contribution of these two cues to task performance because the utilization of those cues taps on observers' abilities that are not directly related to the perceptual faculty that the current study intends to measure in the BM test, i.e., integration of local motion signals into a coherent global percept.

**Figure 3:**
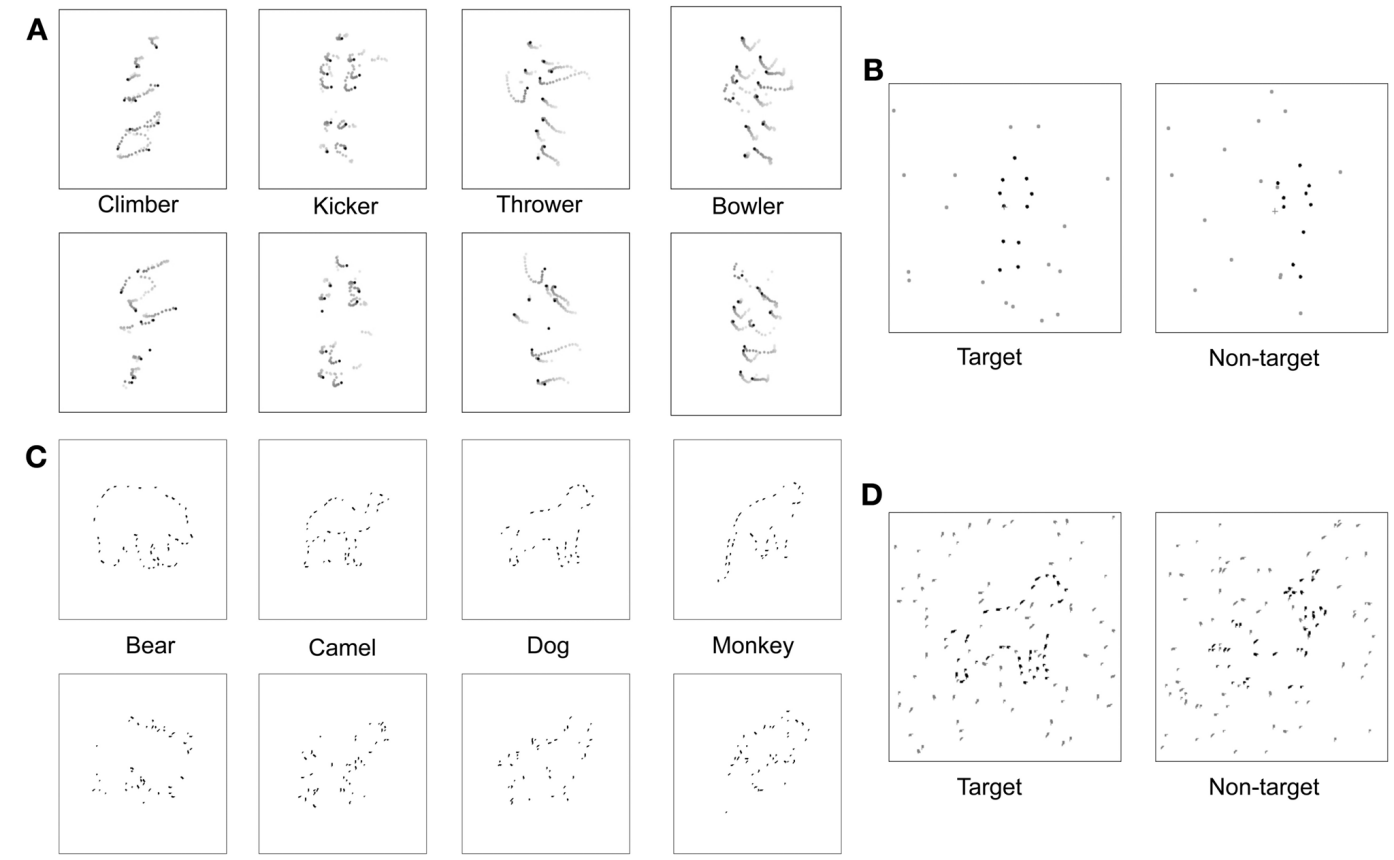
**Visual stimuli. (A)** Space-time plot of changing dot positions associated with each of four PL activities, for both normal animations (top row) and scrambled versions of those animations (bottom row). In each panel the initial dot positions are shown as black dots, and the subsequent trajectory of each dot is shown by the lighter colored dots. **(B)** On each trial the observer viewed two successive PL presentations, illustrated here by a single frame from the first and the second animations. One presentation (first interval in this example) contained a normal PL animation embedded in a predetermined number of noise dots and the other presentation contained a scrambled version of that PL animation also embedded in the same number of noise dots. The number of noise dots varied over trials according to an adaptive procedure that estimated the level of noise producing a criterion level of performance on this 2IFC task. **(C)** Schematic of fragmented figures produced by removing 40% of the total contour length of the outline of an animal; the algorithm for removing portions of a contour was designed to distribute the missing parts roughly equally around the entire shape, with upper and lower limits on the length of removed contour. During the main experiment, degree of fragmentation remained constant, with the number of distracting noise contours varied over trials. **(D)** On each trial the observer viewed two successive presentations, one containing a FF (the target) embedded in noise and the other containing a scrambled version of that FF (non-target) embedded in the same level of noise. The interval containing the target varied randomly over trials, and the level of noise varied according to an adaptive procedure that estimated the level of noise producing a criterion level of performance on this 2IFC task.

In the newly created DM animation sequences, we attempted to minimize these two undesirable cues in the following way. First, we chunked the twelve point light dots into six pairs of dots that represent six body parts, respectively: the head-hip joints, the shoulder joints, the left-upper-limb joints, the right-upper-limb joints, the left-lower-limb joints, and the right-lower-limb joints. Second, we *shuffled (swapped)* the positions of these six dot pairs, instead of randomly repositioning the twelve individual dots, while applying the following constraints: (i) the head-hip joints and the shoulder joints should not be swapped; (ii) the two upper-limb pairs should not be swapped; (iii) the two lower-limb pairs should not be swapped. Third, as in the target interval, the spatial location of a DM sequence was varied unpredictably from trial to trial within the total display extent. Schematic examples of each of the four target and non-target animations are shown in Figure 3A. The chunking and constrained shuffling techniques together helped maintain local motion energy levels across frames of a DM sequence and helped maintain the same overall dot distributions in individual frames of both the DM sequence and the original animation sequence. Unpublished pilot work in our laboratory confirms that most participants find it more difficult to discriminate original BM sequences from their DM counterparts than from their scrambled counterparts, presumably because the residual cues in the scrambled versions are no longer available in the DM versions. Readers are invited to make these comparisons for themselves by viewing the two example video clips in the supplementary materials section—one video depicts an underhand bowling action, shown with reds for purposes of illustration, accompanied by noise dots shown here in black, and the other video clip shows a disordered motion version of this action. In the actual experiments, of course, all dots were black. We are currently preparing an expanded description of these DM animations and comparisons of them to conventional scrambling (Kim et al., in preparation).

On each trial, the two intervals (target and non-target) contained a variable number of noise dots, with the same number appearing on each interval of a given trial (see Figure 3B). Noise dots were generated online (i.e., prior to each trial) by replicating the trajectories of the twelve dots comprising the PL figure, with independent noise samples for the target and non-target intervals. The number of noise dots was varied over trials according to an adaptive procedure that targets the 75% correct level of performance on this 2IFC task. Needless to say, the task became more difficult—and eventually impossible—as the number of noise dots increased. The index of task performance is the noise-to-signal ratio (NSR), which is the ratio between the threshold number of noise dots estimated by Quest and the number of dots comprising the PL figure (12).

#### Procedure

In the main experiment, each participant was first familiarized with the stimuli and task prior to formal testing. During this familiarization phase, a participant began by viewing each of the four BM activities and naming or describing to the experimenter the activity portrayed in the sequence. All participants performed flawlessly on this simple judgment. Next, each participant performed a few 2IFC practice trials in which the target PL figure appeared together with a small number of noise dots. For the initial couple of practice trials, the dots defining the PL figure were colored in red, making the figure stand out from the black noise dots; for the next several practice trials, the PL dots were black. The experimenter carefully explained to the participant that the human figure would appear in only one of two intervals.

Following this familiarization phase, the participant began the formal testing phase, which involved 3 blocks consisting of 50 trials/block. During a block, the participant initiated each trial by depressing the space bar, and after each trial registered his/her response by pressing the “1” or “2” button on a computer keypad to designate which interval contained the target; the participant was not required to identify the activity. At the end of each block, QUEST computed and recorded the estimated threshold (number of noise dots) for 75%-correct discrimination of BM from DM, together with the trial-by-trial record of stimuli presented and responses made for the entire 50-trial sequence.

### Task 2: fragmented figures discrimination

#### Stimuli

The target stimuli used for the FF discrimination task were black outline drawings of animals in which portions of the outline defining the figure were erased. Stimuli like this have been used in a wide variety of other studies (e.g., Snodgrass and Feenan, 1990). The difficulty of recognizing the figure depends on the extent of deletion of the outline contour and, as we have exploited, the presence of noise contours forming the background against which these figures are seen. The animal figures used in our study were 4-limbed mammals in poses where the animal was at rest and most of its limbs were visible. To create the line drawings of these animals, we obtained animal silhouettes from the web, which we then processed in MATLAB to create black on white drawings the outlines of which were 1 pixel in thickness. Ordinarily one would vary the extent of outline deletion to manipulate the ease of figure recognition, but we wanted to make this task formally identical to the BM task where the noise level was varied to manipulate target discriminability. Accordingly, we produced a set of figures all degraded to a given degree and then presented those figures within a background of noise that was varied over trials.

To produce the fragmented animals, we removed 40% of the total visible contour defining the outline of each figure, with each segment of removed contour being anywhere from 7 to 14 pixels in length, sampled randomly from a uniform distribution. Our initial sample included 15 different animal figures that we narrowed to four exemplars based on pilot testing that was identical to that described for the biological motion sequences. For this FF pilot experiment, the starting NSR value was 2, and the staircase was terminated after 16 reversals and the performance threshold was estimated by averaging the NSR present at the last 8 reversals. For FF, the NSR increased by 0.2 per two successive correct trials for the first 12 reversals, then decreased to 0.1 per two successive correct trials in the remainder of the block sequence. Participants performed an average of 983 trials for this FF pilot task. The four figures most similar in discriminability based on pilot testing were bear, dog, monkey, and camel. These four exemplars could also be displayed in left-right reversal, thereby creating a total of 8 distinct test figures. Line segments defining the four animal figures fell within a square area approximately 5° on a side. Examples of each of the four fragmented target figures are shown in the top row of Figure 3C.

To make non-target versions of these figures, we randomly relocated each small, oriented line segment comprising a target animal figure to new positions around the virtual outline of that figure; for example, a short horizontal contour associated with the top of the head could end located at a position previously occupied by a vertical contour associated with part of a leg. Contours could also be spatially jittered to prevent two adjacent contours from connecting to one another. These shuffling maneuvers destroyed the implied contour continuation within local regions of the figure but preserved the overall contour that was also contained within the target version of the figure. Examples of each of the four non-target figures are shown in the bottom row of Figure 3C. The non-target figure (shuffled fragments) was remade online before each trial, but the target figure (unshuffled fragments) was fixed in configuration throughout the experiment. The interval containing the target was randomized over trials, and the target and non-target stimuli were randomly jittered (up to 1.5°) about the central fixation point within the display on each trial interval.

The noise mask in which the target and non-target figures appeared consisted of line segments, which were members of the original complete line drawing of the animal (see Figure 3D). The level of noise mask (noise-to-signal ratio: NSR) presented on each trial was determined by QUEST. The higher the NSR, the more difficult it was to discriminate which interval contained the target animal figure.

### Procedure

In familiarization phase prior to the main experiment, the participant viewed the target stimulus without noise and was asked by the experimenter to verbally indicate whether or not he/she could see “something.” Next, participants performed a few 2IFC trials in which the figure appeared in weak noise, with the fragmented contours defining the target animal colored in red. This step was included to illustrate what the target looks like with a noise mask. Lastly, the participant performed a few practice trials on which all visible contours were black and the NSR was 2.

The formal testing consisted of 3 blocks of the FF task, with 50 trials devoted to each block. Each trial was initiated when the participant pressed the space bar, which triggered presentation of two 1-s display intervals with a 0.5 s blank screen between intervals. Following each trial, participants pressed a button that corresponded to the interval, first or second, containing the target stimulus. At the end of each block, QUEST computed and recorded the noise level associated with 75%-correct performance together with the trial-by-trial record of stimuli presented and responses made for the entire 50-trial sequence.

### Task 3: gabor patch detection

#### Stimuli

The target stimulus, presented during one of two successive 1-s intervals, was a gabor patch: a vertically oriented, 0.3 cycle/degree sinusoidal grating imaged within a circular aperture (diameter = 6.4°) whose borders were blurred by a Gaussian envelope (*SD* = 1.3°). During the target interval, this gabor patch was presented against a noisy gray background (high frequency random dot, grayscale noise); the exact location of the gabor patch was jittered (up to 1°) relative to the central fixation mark, to discourage the participant from monitoring a single location within the display. The non-target interval presented the noise background only.

#### Procedure

Prior to the main experiment, the participant was shown a high contrast example of the gabor patch to familiarize him/her with the stimulus. The participant then performed 9 practice trials (2IFC) that included three levels of gabor contrast (easy, medium, and hard: 0.2, 0.1, and 0.06). In the main experiment, each participant was tested on 3 successive blocks of trials, with each block comprising 50 trials. Within each block the contrast of the gabor target was varied according to QUEST to estimate the level of contrast associated with 75%-correct detection performance. For purposes of correlation analyses, contrast threshold values were converted to sensitivity, which is the reciprocal of contrast threshold (e.g., a threshold of 0.02 corresponds to a sensitivity value of 50).

## Results

**Figure 4:**
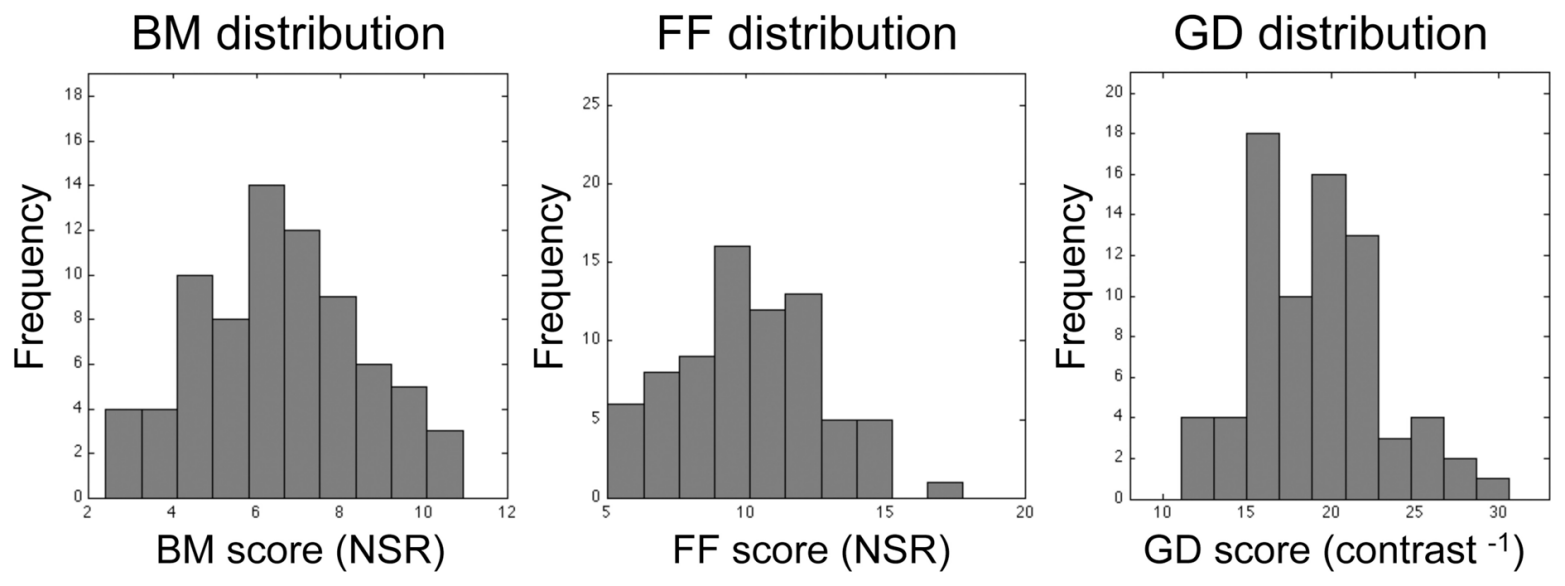
**Histograms showing distributions of threshold estimates associated with each of the three visual tasks.** Bin size was optimized using the procedure recommended by Freedman and Diaconis (1981). Lilliefors test for normality reveals that none of these distributions is statistically significantly different from the normal distribution (*p* = 0.84, *p* = 0.79, and *p* = 0.28 for BM, FF, and GD, respectively).

Among the 76 individuals who successfully completed the study, there was substantial individual variability in performance on all three tasks, as can be seen in the histograms plotted in Figure 4. Within the entire data set, there were a few, meaninglessly low values returned by QUEST because a participant failed to discriminate between target and non-target intervals at the lowest level of noise predetermined by QUEST in a given block of trials. The performance of one participant was remarkably and consistently poor on one task: he performed at chance level (50%) on all three QUEST blocks for the FF task at the lowest noise level tested. Even though this individual understood the instructions and described seeing the degraded figure during the demonstration sequences, and his performance on the other two tasks was unremarkably normal, we decided to eliminate his data from all analyses. With this participant eliminated, a floor effect was seen in only 3 threshold estimates out of the total of 675 estimates (= 3 blocks × 3 tasks × 75 participants) derived from the remaining participants. This effect was observed in one block for each of three different participants. For each of these three blocks we recorded the threshold as zero. The analyses and conclusions described below do not change if those zero values are excluded.

For each task, we implemented the threshold performance score in two ways: (1) the unweighted arithmetic average of the participant's three threshold estimates from his/her three QUEST blocks; and, (2) a weighted average of the three estimates from each QUEST block, whereby assigned weights were inversely proportional to the within-subject standard deviations provided by QUEST. The results and conclusions were equivalent across the two sets of analyses. For the sake of brevity we present the results of the unweighted analyses below.

As can be seen in Table 1, there were no significant differences in performance on any of the three tasks between males and females [*t*_(74) = −1.77_, *p* = 0.08 for BM; *t*_(74)_ = 0.72, *p* = 0.47 for FF; *t*_(74)_ = 0.42, *p* = 0.67 for GD], so for all subsequent analyses we pooled results from both genders. Somewhat surprisingly, participants from SNU performed better than participants from VU on the BM task, *t*_(73)_ = 4.46, *p* < 0.0001 (SNU *x* = 7.11, *SD* = 1.79; VU *x* = 5.03, *SD* = 1.79), and on the FF task, *t*_(73)_ = 2.47, *p* = 0.02 (SNU *x* = 10.45, *SD* = 2.50; VU *x* = 8.83, *SD* = 2.58). Conversely, VU participants out-performed their SNU counterparts on GD, *t*_(73)_ = 2.78, *p* = 0.007 (SNU *x* = 18.44, *SD* = 3.37; VU *x* = 21.15, *SD* = 4.58). Due to these results, we incorporated site (SNU/VU) into the correlation and regression analyses described below.

**Table 1:** **Average threshold by groups**.

	**avg BM**	**avg FF**	**avg GD**
	**(NSR) ± *SD***	**(NSR) ± *SD***	**(contrast^−1^) ± *SD***
SNU (*n* = 55)	7.11 ± 1.79	10.46 ± 2.50	18.44 ± 3.38
VU (*n* = 20)	5.02 ± 1.79	8.83 ± 2.58	21.15 ± 4.58
Female (*n* = 39)	6.17 ± 2.01	10.12 ± 2.61	19.38 ± 3.61
Male (*n* = 36)	6.97 ± 1.94	9.92 ± 2.64	18.93 ± 4.22
Pooled	6.55 ± 2.00	10.03 ± 2.61	19.16 ± 3.90

**Figure 5:**
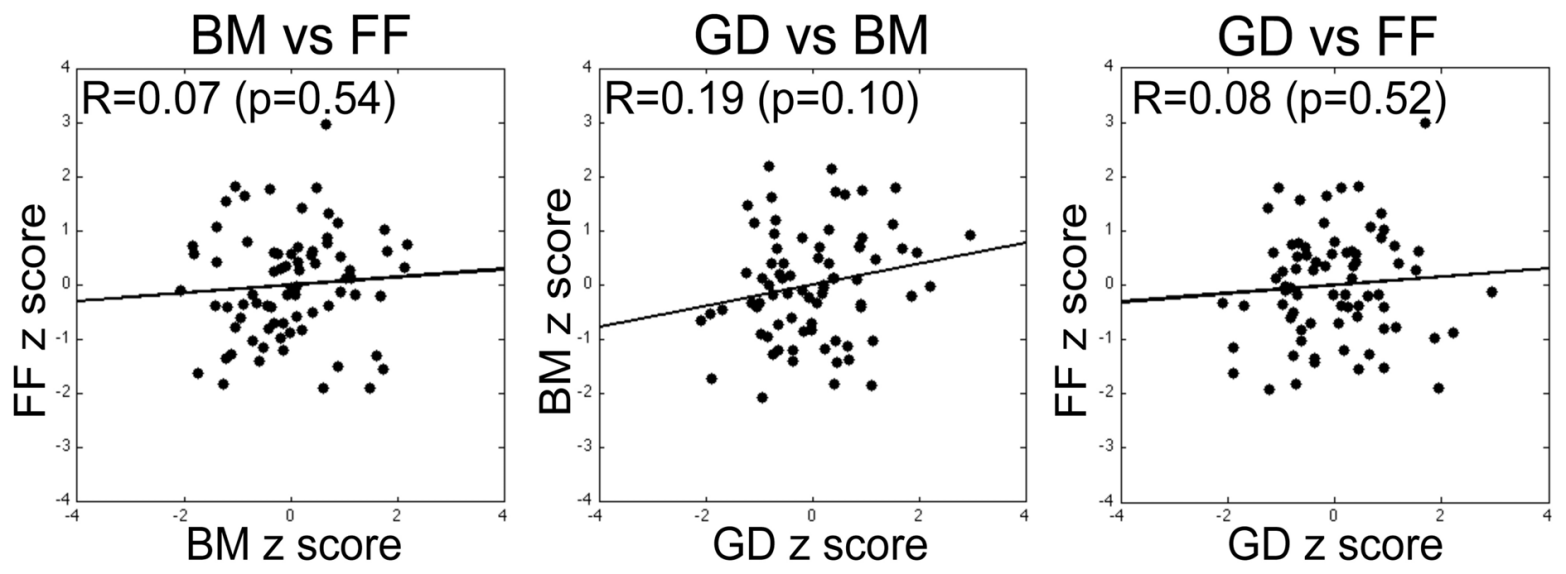
**Correlation between performance measures on pairs of tasks (specific pairs specified by labels above each plot), with each data point within a panel corresponding to a given participant's threshold scores for the two tasks expressed in units of z-score derived by transforming the unweighted threshold estimates for the two tasks (simple average of estimates from the three blocks) into a *z*-score.** None of the pairwise correlations was statistically significant. The same pattern of results was obtained for correlations computed using the raw scores and on correlations computed using the weighted estimates of threshold for the two tasks (see text for details).

For our initial analyses we computed Pearson correlations on all pair-wise combinations of performance scores on the three tasks. Three sets of correlations were performed: (1) Zero-order correlations on the total sample aggregating the data across the two sites (SNU and VU; see Figure 5); (2) Zero-order correlations within each of the two sites; and (3) Partial correlations performed on the aggregated data adjusting for site. The latter estimate the average within-site correlation and thus eliminate the effect on correlations of between-site differences in means. As is evident by the correlations and normal-theory 95% confidence intervals based on *r* to *Z* transformations reported in Table 2, across both sets of zero-order correlations the relation between BM and FF was low and non-significantly different from 0. While FF and GD performance measures were not significantly correlated on either the overall or within-group computations, BM and GD were significantly correlated within both sites and nearly significantly correlated overall. However, several different tests of dependent correlations (e.g., Preacher, 2006; Steiger, 1980) indicated that the BM-GD correlation was not significantly greater than the FF-GD correlation either overall (all *p*s > 0.40) or separately within the SNU (all *p*s > 0.60) and VU (all *p*s > 0.079) groups.

**Table 2:** **Summary of correlations between tasks**.

**Correlation measure**	**BM with FF**	**BM with GD**	**FF with GD**
**PEARSON**			
**Pooled**			
*r*	*r* = 0.07 (*p* = 0.54)	*r* = 0.19 (*p* = 0.09)	*r* = 0.08 (*p* = 0.52)
95% CI	(−0.16 to 0.29)	(−0.03 to 0.40)	(−0.15 to 0.30)
(Normal)			
95% CI	(−0.17 to 0.29)	(−0.02 to 0.39)	(−0.17 to 0.33)
(Bootstrap)			
**SNU**			
*r*	*r* = 0.00 (*p* = 0.99)	*r* = 0.32 (*p* = 0.02)	*r* = 0.23 (*p* = 0.09)
95% CI	(−0.26 to 0.26)	(0.05 to 0.53)	(−0.04 to 0.47)
(Normal)			
95% CI	(−0.28 to 0.26)	(0.05 to 0.52)	(−0.06 to 0.51)
(Bootstrap)			
**VU**			
*r*	*r* = −0.25 (*p* = 0.30)	*r* = 0.59 (*p* = 0.01)	*r* = 0.08 (*p* = 0.76)
95% CI	(−0.62 to 0.22)	(0.20 to 0.82)	(−0.38 to 0.50)
(Normal)			
95% CI	(−0.58 to 0.13)	(0.15 to 0.84)	(−0.34 to 0.44)
(Bootstrap)			
**PERCENTAGE-BEND**			
**Pooled**			
*r*	*r* = 0.11 (*p* = 0.40)	*r* = 0.15 (*p* = 0.16)	*r* = 0.06 (*p* = 0.66)
95% CI	(−0.16 to 0.34)	(−0.08 to 0.38)	(−0.20 to 0.28)
(Bootstrap)			
**SNU**			
*r*	*r* = 0.07 (*p* = 0.68)	*r* = 0.31 (*p* = 0.03)	*r* = 0.16 (*p* = 0.28)
95% CI	(−0.24 to 0.38)	(0.03 to 0.54)	(−0.14 to 0.42)
(Bootstrap)			
**VU**			
*r*	*r* = 0.-0.28 (p =	*r* = 0.52 (p = 0.04)	*r* = 0.03 (*p* = 0.85)
95% CI	0.28) (−0.62 to 0.26)	(0.01 to 0.85)	(−0.42 to 0.49)
(Bootstrap)			
**SPEARMAN**			
**Pooled**			
*r*	*r* = 0.12 (*p* = 0.31)	*r* = 0.17 (*p* = 0.16)	*r* = 0.05 (*p* = 0.68)
95% CI	(−0.11 to 0.34)	(−0.07 to 0.38)	(−0.18 to 0.27)
**SNU**			
*r*	*r* = 0.10 (*p* = 0.48)	*r* = 0.29 (*p* = 0.03)	*r* = 0.16 (*p* = 0.24)
95% CI	(−0.17 to 0.35)	(−0.17 to 0.35)	(−0.10 to 0.41)
**VU**			
*r*	*r* = −0.25 (*p* = 0.29)	*r* = 0.42 (*p* = 0.06)	*r* = 0.07 (*p* = 0.78)
95% CI	(−0.63 to 0.21)	(−0.02 to 0.73)	(−0.39 to 0.50)

*Pooled N = 75; SNU N = 55; VU N = 20. Normal theory confidence intervals computed via r to Z transformation. One thousand samples were generated for each bootstrap confidence interval. Pearson bootstrap confidence intervals were computed using the adjusted bootstrap percentile (BC_a_) method. Percentage bend bootstrap confidence intervals were computed using the percentile method*.

The results of the partial correlation analyses were consistent with those of the zero-order correlations. The correlation between BM and FF adjusting for site was extremely low in absolute magnitude and non-significantly different from 0 (partial *r* = −0.07, *p* = 0.58, 95% CI = −0.29 to 0.17). While FF and GD were not significantly correlated (partial *r* = 0.18, *p* = 0.13, 95% CI = −0.06 to 0.39), BM and GD were significantly correlated (partial *r* = 0.40, *p* = 0.0004, 95% CI = 0.19 to 0.57). However, similar to the zero-order correlational analyses, the BM-GD partial *r* was not significantly greater than the FF-GD partial *r* when tests were conducted within a structural equation modeling framework (Preacher, 2006) using MPLUS software (Muthén and Muthén, 1998–2011), χ ^2^_(1)_ = 2.05, *p* = 0.15.

The fact that the correlations between GD and BM were consistently either significant or nearly significant raised the possibility that the magnitude of the relation between BM and FF might change somewhat if we mutually adjusted for GD. From a conceptual perspective such adjustment assesses the relation between BM and FF removing the influence of motivational factors on task performance. In fact, such adjustment had no impact. Whether computed overall or separately within site, the partial correlations between BM and FF adjusting for GD (computed both overall and within-site) were non-significant just as were the zero-order values (all *p*s > 0.13). These results are not surprising given the weak relation observed between GD and FF. The partial correlation between BM and FF adjusting for both GD and site was actually negative, though non-significant, and similar to the partial correlation between the two variables adjusting for site alone (partial *r* = −0.15, *p* = 0.20, 95% CI = −0.37 to 0.08).

We also tested whether the relations among pairs of variables differed across the two sites. It is commonly recommended that such comparisons be conducted on unstandardized regression coefficients because between-group differences in correlations or other standardized measures can be observed simply due to differences in variances and covariances across groups (e.g., Duncan, 1975; Kim and Ferree, 1981). We conducted multiple regression analyses with the measure of interest (e.g., GD) serving as the dependent variable and three predictors: the other measure of interest (e.g., FF), a dummy variable (coded 0 = SNU and 1 = Vanderbilt) denoting site, and an interaction term formed by the product of the continuous predictor and the dummy variable. Of critical interest was the interaction term that directly estimated differences in regression coefficients across the two sites (Aiken and West, 1991). We conducted six analyses in all, with each task (e.g., GD) serving as a predictor of performance on each of the two remaining tasks (e.g., FF and GD). All interaction terms were non-significant (all *p*s > 0.07) (Table 3). Thus, there was no evidence that the relations among tasks differed across the sites.

**Table 3:** **Within-site regression coefficients and site × predictor interactions**.

**Dependent measure**	**Predictor**	**SNU B (*n* = 55)**	**VU B (*n* = 20)**	**Predictor × Site Interaction B**	**Interaction *t*_(72)_**
BM	FF	0.001	−0.17	0.17	*t* = 0.92
		(0.10)	(0.16)	(0.18)	*p* = 0.36
FF	BM	0.002	−0.36	0.36	*t* = 0.95
		(0.19)	(0.32)	(0.38)	*p* = 0.34
BM	GD	0.17^*^	0.23^**^	−0.06	*t* = −0.58
		(0.07)	(0.08)	(0.11)	*p* = 0.56
GD	BM	0.59^*^	1.49^***^	−0.90	*t* = −1.79
		(0.26)	(0.43)	(0.50)	*p* = 0.078
FF	GD	0.17	0.04	0.13	*t* = 0.79
		(0.10)	(0.13)	(0.16)	*p* = 0.43
GD	FF	0.31	0.13	0.18	*t* = 0.46
		(0.20)	(0.33)	(0.38)	*p* = 0.65

*All regression coefficients are unstandardized. SNU and VU columns display within-site regression coefficients. Standard errors are shown in parentheses*.

* p < 0.025

** p < 0.01

*** *p < 0.001*.

In order to evaluate the validity of the correlation and regression analyses summarized above, it was important to evaluate whether analytic assumptions were met and to compare alternative procedures with less restrictive assumptions. It is well-known that violations of normality assumptions can distort estimates, confidence intervals and hypothesis tests of correlations (for a review, see Wilcox, 2012). Shapiro-Wilks tests of both the univariate normality (Shapiro and Wilk, 1965) of the three variables of interest (BM, FF, and GD) and their pairwise bivariate normality (Villasenor Alva and Estrada, 2009) were clearly non-significant (all *p*s > 0.35), thus indicating close correspondence to normality. Nevertheless, for each pair of variables and subgroup (sample as a whole, SNU, VU), we computed correlations across 1000 bootstrapped samples. We then used several different methods (e.g., bootstrap percentile, adjusted bootstrap percentile, approximate bootstrap) to compute 95% confidence intervals around correlation coefficients (for a review, see DiCiccio and Efron, 1996). In all cases, the confidence intervals and the pattern of non-significant and significant results (e.g., intervals that did and did not encompass 0) corresponded very closely to values generated by the standard normal-theory approach (see Table 1 for one representative set of results yielded by the adjusted bootstrap percentile (BC_a_) method). It is most important to note that all confidence intervals for the correlation between BM and FF included 0.

Pearson correlations and ordinary least squares linear regressions are both particularly sensitive to outliers, which can distort coefficients relative to their true values (Wilcox, 2001, 2012). To address this issue, in accord with the recommendations of Pernet et al. (2012; see also Rousselet and Pernet, 2012), we computed both skipped-correlations (Wilcox, 2004) and percentage-bend correlations (Wilcox, 1994) on the complete data and separately within the SNU and VU groups. The former procedure computes Pearson correlations after detecting and eliminating outliers in bivariate space while the latter down-weights a specified percentage of observations that deviate from the medians of the marginal (i.e., univariate) distribution of each variable. In both cases, the confidence interval bounds and pattern of non-significant and significant findings were completely consistent with those of the Pearson correlations (see, e.g., the percentage-bend results in Table 1).

For comparison to the partial correlation and linear regression analyses summarized above, we also conducted robust regression analyses using the S (Rousseeuw and Yohai, 1984) and MM estimation (Yohai, 1987) procedures, both of which correct estimates for the effects of outliers. The results directly paralleled the un-corrected analyses summarized above. BM and FF were not significantly related when we adjusted for between-site differences in performance (*p*s > 0.67). Similarly, the interactions between group and each of these two variables were non-significant (*p*s > 0.30).

Could the linearity assumption underlying Pearson zero-order and partial correlation coefficients be contributing to our failure to uncover any relation between BM and FF? To address that possibility, we computed Spearman rank order correlations that only assumed monotonicity and not linearity. Neither the Spearman *r*s computed by aggregating the data across groups or separately within groups produced any significant correlations between BM and FF (overall *r* = 0.12, *p* = 0.31; SNU *r* = 0.10, *p* = 0.48; VU *r* = −0.25, *p* = 0.28; see Table 2 for confidence intervals).

In addition, to test for monotonicity while adjusting for any between-group differences in means, we computed a spline analysis with monotonicity constraints. Relative to global polynomials, splines are more sensitive to local variations in the functional form between two variables and thus typically better at capturing complex or subtle non-linear relations (Ruppert et al., 2003). The monotonicity constrained spline analyses that we conducted used B-spline cubic transformations of the data and either three, five, or seven equally spaced knots. We predicted performance on a given task (either BM or FF) by a spline-transformed representation of the other task of interest and a dummy variable denoting site (in order to remove the effects of the between-site differences in means). In all cases, statistical tests of the monotonic spline fit indicated that a given task failed to significantly predict performance on the other task (all *p*s > 0.23).

We then assessed whether the two tasks were related in a non-monotonic fashion. To check this possibility, we conducted two types of analyses. First, we computed a series of polynomial regression analyses including progressively higher-order terms from 2nd order (i.e., quadratic) up to 8th order. To adjust for between-site differences in means we also included a dummy variable denoting site. No polynomial of any order produced a statistically significant improvement in fit above and beyond a model that included site alone as a predictor of performance (all *p*s > 0.14). We also computed a series of spline analyses that did not impose any monotonicity constraints. Natural cubic splines were used that have optimal smoothness properties among all interpolating functions (e.g., Wood, 2006). We conducted natural cubic spline analyses predicting BM from FF and vice versa with numbers of knots varying from 3 to 10. We also included a dummy variable denoting site in order to account for mean differences. None of the spline components provided a statistically significant and satisfactory fit to the data above and beyond a model that included only site as a predictor (all *p*s > 0.15). In addition, the Akaike Information Criterion (AIC; Akaike, 1973) used for model selection favored a model that included no spline terms and only an intercept term and the dummy variable denoting site. Identical conclusions were reached when a generalized additive modeling (GAM) framework (Hastie and Tibshirani, 1990; Wood, 2006) was used to specify penalized splines and conduct model comparisons and when additional spline models were specified that allowed for different spline functions within the two sites.

One might argue that the null findings concerning the association between BM and FF were due to sample sizes that limited statistical power and precision. While this might be true to some degree for the small subset of analyses conducted within the VU subgroup (*N* = 20), power calculations indicated that the total sample size of 75 is clearly sufficient to reveal effects of a magnitude that are noteworthy. For example, with this sample size, the power to reject the null hypothesis that ρ = 0 is at least 0.80 when the population correlation is greater than or equal to 0.32. Similarly, in the context of partial correlation and regression analyses including both site and a given performance measure as predictor of another measure, our power was approximately 0.80 to detect an increment in the R^2^ due to the performance measure of 0.07 or greater. This increment corresponds to a small to medium effect according to Cohen (1988). Note also we had sufficient power to detect statistically significant correlations between the BM and GD tasks on even the within-site analyses. Moreover, when the question of interest is the magnitude of the relation between two variables, precision of estimation is likely a more important consideration than the power of hypothesis tests. The confidence intervals shown in Table 2 and in the text are a direct reflection of the precision linked to our sample size. In the context of the relation between BM and FF the most important point to note is that even the *upper bounds* of the confidence intervals indicated at best only a weak relation between the two variables (e.g., upper bound for zero-order Pearson *r* = 0.28, upper bound for partial *r* adjusting for site = 0.17).

Because the reliability of a measure constrains the magnitude of the correlation that it can have with another measure (Nunnally and Bernstein, 1991), we addressed whether the non-significant correlations that we observed between BM and FF were due to measurement error. For each task we computed intraclass correlations coefficients (ICCs; Shrout and Fleiss, 1979) based on scores from each of the three blocks that we averaged to form the scores used in the correlation and regression analyses that we had conducted. There are several variants of the ICC. The one that we computed allowed us to estimate the consistency (i.e., reliability) of the average performance measures derived by aggregating across blocks. The estimated ICC's for the average scores were 0.51 for BM and 0.43 for FF (both *p*s < 0.01). Thus, an estimated 51% of the total variability in performance on BM and estimated 43% of the total variability in performance on FF reflected reliable individual differences. Although these values are lower than would be expected for measures of individual differences in the domain of personality assessment, they do indicate that a large proportion of performance on the across-block averages that we used for the correlation and regression analyses reflected reliable individual differences. Interestingly, the ICC for GD (0.66; *p* < 0.01) was even higher. This is very likely because the GD task, unlike BM and FF, did not involve introduction of random, variable strength noise on each trial. Thus, the GD task required simple detection and not recognition of form in noise.

Because lack of reliability can constrain the magnitude of correlations between variables, we computed correlations between BM and FF that corrected them for the attenuating effect of measurement error (Charles, 2005). Although estimated correlations were almost exactly doubled in size relative to the non-corrected ones shown in Table 2, none were statistically significant (all *p*s > 0.25). We also conducted regression analyses that corrected coefficients for attenuation due to measurement error. Using MPLUS software (Muthén and Muthén, 1998–2011), we created latent variables for BM and FF that were corrected for measurement error using the reliability estimates obtained from the ICC analyses. We regressed one latent variable (e.g., BM) on the other one of interest (e.g., FF) and additionally specified paths from a dummy variable denoting site to both measures. By this means, we estimated pooled within-group regression coefficients that were corrected for unreliability of the performance measures. The regression coefficients were not significantly different from zero (*p* = 0.57).

We also examined whether response biases might have affected the estimated relations between BM and FF. We computed the proportion of trials in which each participant chose the first or second interval as containing the target stimulus. Because the target and non-target intervals were randomized across trials the expected proportion of trials on which the target actually appeared was 0.50 for each interval. On both tasks participants demonstrated a slight though statistically significant tendency to choose the first interval [BM*X* = 0.59, for null Ho: *p* = 0.50 *t*_(74)_ = 12.41, *p* < 0.001; FF*X* = 0.55, for null Ho: *p* = 0.50 *t*_(74)_ = 6.81, *p* < 0.001]. The two response biases were weakly correlated (Pearson *r* = 0.20, *p* = 0.086, Spearman *r* = 0.23, *p* = 0.045). We also computed a partial correlation between performance (i.e., threshold) measures of BM and FF while adjusting simultaneously for measures of response bias on each task. This partial correlation was not significant (*r* = 0.08, *p* = 0.51) and almost identical to the zero-order correlation reported in Table 1 (*r* = 0.07, *p* = 0.53). In addition, multiple regression analyses indicated that measures of response bias failed to moderate the relation between the two sets of performance measures (threshold × bias interaction *p*s > 0.80).

In sum, we tested various hypothetical relations between BM and FF, which differed in terms of linearity, monotonicity, and sensitivity to outliers and response biases. In all cases we found no evidence indicating a significant relation between measures of performance on these tasks. Null findings were further observed when we corrected correlations and regression coefficients for the effects of measurement error. These results forced us to conclude that individual differences in BM and FF task performance are largely unrelated.

## Discussion

We tested a large group of young adults on three different perceptual tasks, two of which require integration of visual information (BM and FF) and a third (GD) that involves detection of a simple 1D pattern. Our focus was on the potential correlation in performance between BM and FF, with the GD task included to evaluate a possible contribution from non-sensory factors (e.g., individual differences in general level of motivation) that could have produced a significant correlation between the two tasks of interest. Our results disclose that there are indeed measurable individual differences in the performance on all three tasks, an outcome that was not unexpected given the growing body of evidence documenting the range of performance scores among normal participants tested on a variety of visual tasks (Simpson and McFadden, 2005; Kanai et al., 2010; Schwarzkopf et al., 2010; Genç et al., 2011; Hancock et al., 2012) including perception of biological motion (Heenan and Troje, 2012; Miller and Saygin, 2013). Of course, a wide range of performance scores among participants is desirable, for it sets the stage for assessing correlations between performance scores on the two tasks of interest, i.e., BM discrimination and FF discrimination. Despite the range of individual differences measured on our tasks, however, there was no significant correlation between performance scores on BM and FF. The null findings were consistent across standard Pearson correlations and ordinary least squares regression analyses, robust and non-parametric alternatives, spline and higher-order polynomial analyses designed to detect subtle non-linear relations, and calculations of correlations that were corrected for reliability attenuation due to measurement errors. Moreover, our power calculations indicate that our sample size was sufficiently large to detect correlations of a non-trivial magnitude. Although we certainly cannot and do not claim that the true correlation between these tasks is zero, the confidence intervals around our observed correlations indicate that, at best, the correlation is very low. In the following paragraphs, we consider what this lack of correlation may be telling us about the nature of these two tasks and their underlying neural bases.

Focusing first on the abilities being tapped by BM and FF, the two categories of stimuli have in common the property that a figure, either a human (BM) or an animal (FF), is defined by spatially distributed tokens: dots of light in the case of BM and short, oriented contours in the case of FF. For both types of stimuli, consequently, local features must be integrated spatially in order for the global figure to be discerned. This similarity was one reason why we speculated that performance on the two tasks might be related. But in another respect, the two categories of stimuli are markedly different, and this may provide the key to understanding the absence of correlation in our measures of perception of those stimuli. Specifically, the contour segments defining the FFs are stationary during the display presentation (i.e., the figural elements are static), but for BM the dots defining the PL figure move along hierarchical, pendular paths that portray the kinematics of limb motion (i.e., the tokens are dynamic). Now it is true that form information, under some circumstances, can contribute to perception of BM (Beintema and Lappe, 2002; Beintema et al., 2006; Hiris, 2007; Thirkettle et al., 2009), but there is debate concerning the manner and extent of that contribution (Giese and Poggio, 2003; Casile and Giese, 2005; Lange and Lappe, 2006; Lange et al., 2006; Thurman et al., 2010). Without a doubt, however, motion adds to the salience of BM produced by PL animations (Johansson, 1973). Indeed, a recent study combining psychophysics with a feature extraction algorithm confirms that local motion signals provide the salient ingredient for perception of biological motion from PL animations (Thurman and Grossman, 2008). This conclusion is consistent with other, earlier studies that pinpointed movements of certain parts of the body as being particularly important, by removal of selected dots from PL animations (Mather et al., 1992) or by masking and inversion that perturbs PL perception (Chang and Troje, 2009) Moreover, because of their dynamic nature, PL animations can also portray 3D information about perspective (e.g., De Lussanet et al., 2008) and occlusion (Jackson and Blake, 2010), something not necessarily available in static, fragmented line drawings, i.e., FFs.

Now to be clear, we are not asserting that form information plays no role in BM perception for, as mentioned above, there are reasons for thinking it does. We are suggesting, however, that these two visual abilities—perceiving FFs and perceiving BM—are distinctively different in terms of their dependence on visual motion. This means, in turn, that the processes involved in global form integration of the sort engaged by the FF task are likely not the same as those responsible for discerning the presence of BM in dynamic noise, even though superficially both entail perceptual organization and recovery of a meaningful object within noise. Of course, in addition to the BM and FF target stimuli, there are substantial differences, too, in the masking noise arrays used in this study. Specifically, BM noise comprised dots that moved in trajectories identical to those defining the PL figure presented on a given trial, with those noise dots being repositioned randomly within the display area. The FF noise comprised short line segments sampled from the segments defining the FF target presented on a given trial, with those noise segments then repositioned within the display area. Importantly, the BM noise dots were dynamic and the FF noise segments were not. Perhaps these two different forms of noise encouraged observers to adopt different strategies when trying to segment target elements from noise elements in FF and in BM, strategies that are unrelated to one another and, therefore, uncorrelated in effectiveness across individuals.

It is natural to wonder whether the two tasks may be tapping into separate neural mechanisms that are differentially selective for dynamic 3D information vs. static, 2D information (e.g., Orban et al., 2000). Thinking along these lines, it is tempting to turn to the popular notion of dorsal and ventral stream pathways that putatively subserve distinct roles in the processing of visual information supporting motion perception, form recognition, and visually guided actions (Mishkin et al., 1983; Goodale and Milner, 1992; Nassi and Callaway, 2009; Kravitz et al., 2011; Mather et al., 2013). Brain imaging studies using fMRI have found that successful shape perception and object recognition are accompanied by activation of a swath of visual areas comprising the ventral stream, stretching from occipital cortex to temporal cortex and beyond (e.g., see review by Kanwisher et al., 1997). One portion of this pathway, known as the lateral-occipital complex (LOC), has been implicated in object recognition because it responds strongly to objects and line drawings (Grill-Spector et al., 2001) but not to scrambled line drawings (Malach et al., 1995; Kanwisher et al., 1996; Lerner et al., 2002). Pointing to the same conclusion are results from event-related potential measurements in humans that reveal neural responses bilaterally within the occipito-temporal area in response to FFs much like the ones used by us, suggesting a possible neural correlate of perceptual filling in of fragmented objects (Doniger et al., 2000). Complementing those findings are results showing impairments in object recognition in patients with damage to brain areas that include LOC, including tasks requiring integration of local features (Riddoch and Humphreys, 1987; Behrmann and Kimchi, 2003). Called integrative agnosia, this appears to be a hallmark symptom of damage to the infero-temporal cortex. In view of these lines of evidence, it seems reasonable to presume that performance on a FF task would rely heavily on neural mechanisms within the ventral stream.

Motion processing, in contrast, is distributed across visual areas comprising the putative dorsal stream network, areas that include middle temporal area (MT), medial superior temporal area (MST) and posterior region of the superior temporal sulcus (STSp). Moreover, lesion studies underscore the importance of dorsal stream areas in the registration of information supporting perception of BM from PL animations (Saygin, 2007; Pavlova, 2012), and temporary “lesions” induced using transcranial magnetic stimulation applied to area STSp point to the same conclusion (Grossman et al., 2005). At the same time, however, we know that PL animations also activate ventral stream areas including the dorsal posterior region of the LOC and the fusiform face area (Grossman and Blake, 2002; Santi et al., 2003; Grossman et al., 2004; Peelen et al., 2006; McKay et al., 2012). Also selectively activated by biological motion are two visual areas—the ventroposterior visual area (Servos et al., 2002) and the kinetic occipital area (Santi et al., 2003)—that are thought to be involved in the analysis of form. All things considered, it would be premature at this stage to attribute mechanisms associated with perception of BM to one visual stream or the other.

Why did GD thresholds correlate weakly but significantly with BM thresholds but not with FF thresholds? Besides the possibility that FF and BM engage separate neural mechanisms, it could be that the BM and FF tasks confront an observer with different task demands that encourage distinct processing strategies of the sort discussed several paragraphs earlier. According to this speculation, the strategy one tends to employ when trying to detect PL animations is more like that used when trying to detect a gabor patch, compared to the strategy used when confronted with a fragmented animal figure. It is known that the ability to perceive FF follows a more prolonged developmental trend than does the ability to perceive BM: by 9 years of age, children have achieved adult levels of performance at detecting BM in noise (Friere et al., 2006), but adult-like performance on contour integration in noise is not realized before the age of fourteen (Kovács et al., 1999). Contrast sensitivity, the ability tapped by the GD task, achieves adult levels by age eight (Bradley and Freeman, 1982). Of course our participants were young adults, not children, but still the differences in maturational time-lines of BM and FF are suggestive of differences in the neural embodiment of the processes engaged by the two categories of visual stimuli, with GD being more similar to BM than to FF. All that being said, it is important not to over-interpret the pattern of significant vs. non-significant correlations here because we consistently failed to find that the BM/GD correlations were significantly greater than the FF/GD correlations. Our overall pattern of findings indicates that any differences in the overall degree of coherence between GD and BM and FF, respectively, are likely to be fairly subtle.

## Conclusion

This study sought to learn whether the ability to perceive FFs was related to the ability to perceive BM portrayed in PL animations. A variety of different statistical analyses of performance measures from a large sample of young adults consistently failed to disclose a significant correlation between performance on these two tasks. Despite the superficial similarity of the tasks, spatial integration entailed in perception of FFs evidently depends on processes different from those involved in spatio-temporal integration required for perception of BM. In other words, the factors that promote substantial individual differences in perceptual expertise on these two tasks must be operating largely independently. It remains to be learned just what those factors are.

### Conflict of interest statement

The authors declare that the research was conducted in the absence of any commercial or financial relationships that could be construed as a potential conflict of interest.
